# Effect of Nb on the Microstructure, Mechanical Properties, Corrosion Behavior, and Cytotoxicity of Ti-Nb Alloys

**DOI:** 10.3390/ma8095287

**Published:** 2015-09-09

**Authors:** Mi-Kyung Han, Jai-Youl Kim, Moon-Jin Hwang, Ho-Jun Song, Yeong-Joon Park

**Affiliations:** Department of Dental Materials and Medical Research Center for Biomineralization Disorders, School of Dentistry, Chonnam National University, Gwangju 500-757, Korea; E-Mails: mikihan2@naver.com (M.-K.H.); abcdq0111@hanmail.net (J.-Y.K.); mjhwang@jnu.ac.kr (M.-J.H.); songhj@jnu.ac.kr (H.-J.S.)

**Keywords:** Ti-xNb alloys, microstructure, mechanical properties, cytotoxicity, dental material

## Abstract

In this paper, the effects of Nb addition (5–20 wt %) on the microstructure, mechanical properties, corrosion behavior, and cytotoxicity of Ti-Nb alloys were investigated with the aim of understanding the relationship between phase/microstructure and various properties of Ti-xNb alloys. Phase/microstructure was analyzed using X-ray diffraction (XRD), SEM, and TEM. The results indicated that the Ti-xNb alloys (x = 10, 15, and 20 wt %) were mainly composed of α + β phases with precipitation of the isothermal ω phase. The volume percentage of the ω phase increased with increasing Nb content. We also investigated the effects of the alloying element Nb on the mechanical properties (including Vickers hardness and elastic modulus), oxidation protection ability, and corrosion behavior of Ti-xNb binary alloys. The mechanical properties and corrosion behavior of Ti-xNb alloys were found to be sensitive to Nb content. These experimental results indicated that the addition of Nb contributed to the hardening of cp-Ti and to the improvement of its oxidation resistance. Electrochemical experiments showed that the Ti-xNb alloys exhibited superior corrosion resistance to that of cp-Ti. The cytotoxicities of the Ti-xNb alloys were similar to that of pure titanium.

## 1. Introduction

Ti has received widespread attention due to its favorable mechanical properties, including high specific strength, good corrosion stability, and good biocompatibility after implantation [1]. However, Ti has inherent difficulties in being cast accurately due to its high melting temperature (1668 °C) and high reactivity with gases at high temperature. Ti as an implant material has high Young’s modulus, which leads to the stress shield effect brought about by Young’s modulus mismatch between Ti implant material (110~120 GPa) and human bone (~30 GPa). Also, biofluid containing chloride ions or fluoride ions gradually destroy the passive oxide layer formed on Ti. In order to enhance the castability, mechanical properties, and corrosion resistance of Ti, Ti-based alloys have been extensively developed [2]. Among the Ti-based alloys, Ti-Nb alloys have been reported to have a low Young’s modulus, high strength, and good biocompatibility [3,4]. It is known that Nb as a β stabilizing element plays an important role in the lowering of Young’s modulus for titanium alloys [5,6]. Moreover, alloys with lower Nb content exhibit the shape memory phenomenon, which can be used in dentistry for orthodontic wires [7,8,9]. Enhanced corrosion resistance of Ti-Nb alloys in a fluoride environment has been reported [10,11,12], and no evidence of cytotoxicity of Nb-containing dental alloys has been reported to date.

Phase transformation in Ti-Nb alloys has been extensively reported in the literature [3,13,14]. It is known that phase transformation and microstructural morphology are critically influenced by alloy composition and cooling rate variations. Detailed microstructural analysis using TEM and XRD can provide a basis for understanding the anomalous properties of Ti-Nb alloys. Despite the fact that there exists extensive literature on the fundamental physical metallurgy aspects of Ti-Nb alloys, studies assessing the effect of Nb metal on the mechanical properties of titanium are still relatively rare, and hence further investigations are necessary to provide an in-depth understanding of these mechanical properties. 

In the present study, binary Ti-xNb alloys (x = 5, 10, 15, and 20 wt %) were prepared using an arc melting furnace. After homogenization heat treatment of Ti-xNb alloys, the alloys were subjected to slow furnace cooling to 600 °C and air cooling to room temperature in order to obtain α + β phases. The present investigation focused on the relationship between mechanical properties and phase/microstructure of a series of Ti-xNb alloys. The corrosion behavior and cytotoxicity of Ti-xNb alloys were evaluated for compatibility as biomaterials. Henceforth in this work, “Ti-xNb” will stand for “Ti-x wt% Nb”.

## 2. Results and Discussion

### 2.1. Effects of Nb Concentration on Phase and Microstructure

**Figure 1:**
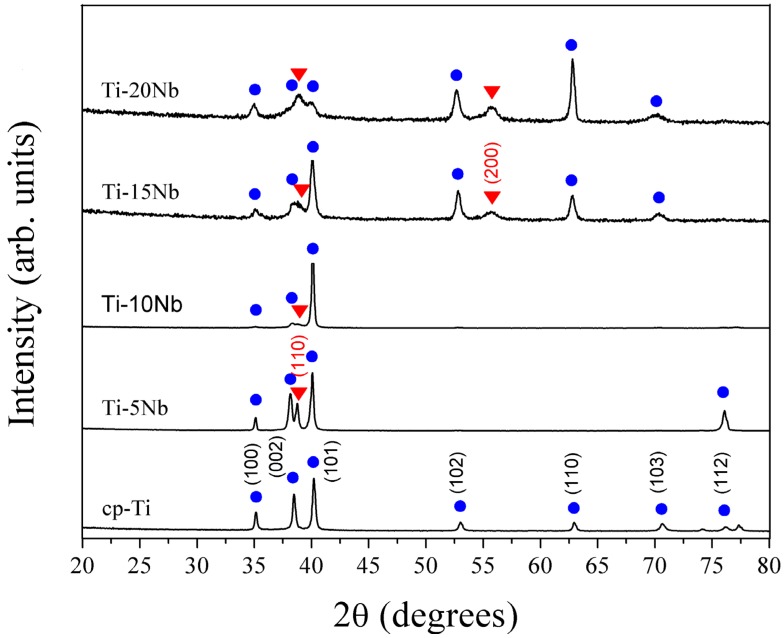
XRD patterns of cast cp-Ti and the series of binary Ti-xNb alloys. The hexagonal Ti and cubic β-Ti peaks are marked with • and ▼ symbols, respectively.

Using transmission electron microscopy (TEM), selected area electron diffraction (SAED) pattern analysis, and X-ray diffraction (XRD) analysis, the phases of cast alloys were characterized. The phases were identified by matching each characteristic peak with the JCPDS files (JCPDS card No.44-1294 for α-Ti) [15]. Figure 1 displays the XRD patterns for a series of Ti-xNb alloys compared with cp-Ti. The phase boundaries were in good agreement with the existing phase diagram [16]. The XRD patterns of all alloys indicated the coexistence of both α and β phases. For the Ti-5Nb alloy, the main phase constituent was the α phase. The presence of Nb in the Ti-5Nb alloy caused an increase in lattice parameters of the α phase (P6_3_/*mmc*, *a* = 2.9554(1), *c* = 4.7207(1) Å). The primary α phase peak increased as Nb content increased to 10 wt %. When Nb content increased over 10 wt %, the α phase peak diminished gradually. It was reported that both the β phase and the ω phase were formed in the binary Ti-Nb systems with Nb content of 12–32 wt %, and the ω phase had a highly distorted transitional hexagonal structure [17]. However, the possible presence of the ω phase was not detected in the XRD profile due to the detection limit of the XRD instrument. The ω phase was easily detected by TEM and is discussed in greater detail in later sections.

**Figure 2:**
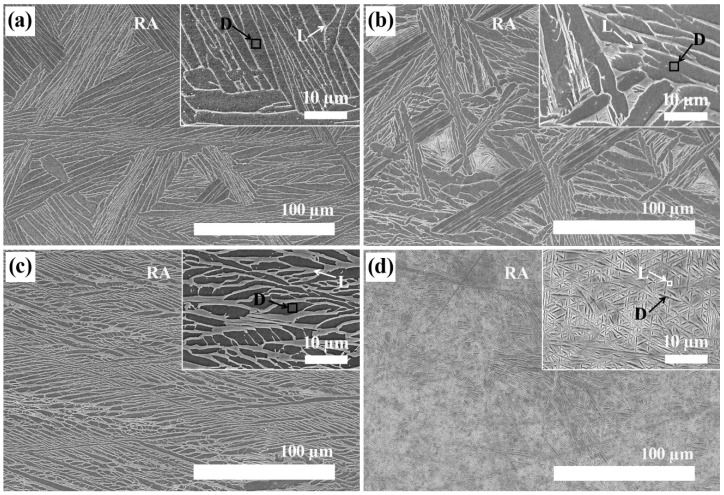
Low-magnification and higher-magnification (inset) SEM images of (**a**) Ti-5Nb; (**b**) Ti-10Nb; (**c**) Ti-15Nb; and (**d**) Ti-20Nb alloys. (RA: rectangular area, 250 µm × 170 µm, D: dark region, L: light region).

The SEM images of Ti-xNb with differing Nb content (5, 10, 15, and 20 wt %) are shown in Figure 2. Microstructural examination showed that significant amounts of β phase existed in all samples, and this finding was in agreement with phase identification by XRD. As can be seen in Table 1, the light and dark regions displayed the Nb-rich phase and the Ti matrix, respectively, in SEM images of polished surfaces shown in Figure 2a–d. The Ti matrix and the Nb-rich phase were analyzed using an energy dispersive X-ray (EDX) detector attached to the SEM. The Ti-5Nb alloy exhibited typical lath type morphology, while the Ti-20Nb alloy showed an entirely acicular structure. In the selected rectangular area (RA), the Nb weight percentage in the Ti-xNb alloys was similar to the nominal wt % of Ti-xNb alloys (Table 1). As the content of Nb in Ti-xNb alloys increased, the weight percentages of Nb in the light region increased from 12.4 to 27.7 wt %, and those in the dark region increased from 3.6 to 8.9 wt %.

**Table 1:** EDX (energy dispersive X-ray) analysis results obtained from selected areas in Figure 2 (*n* = 5).

Element	Weight Percentage (wt%)											
	Ti-5Nb			Ti-10Nb			Ti-15Nb			Ti-20Nb		
	RA	D	L	RA	D	L	RA	D	L	RA	D	L
Ti	94.7 (0.2)	96.4 (0.5)	87.6 (0.8)	89.1 (0.4)	95.3 (0.7)	77.3 (1.4)	83.7 (0.4)	94.4 (0.5)	73.1 (0.7)	79.1 (0.7)	91.1 (0.8)	72.3 (0.7)
Nb	5.3 (0.2)	3.6 (0.5)	12.4 (0.8)	10.9 (0.4)	4.7 (0.7)	22.7 (1.4)	16.3 (0.4)	5.6 (0.5)	26.9 (0.7)	20.9 (0.7)	8.9 (0.8)	27.7 (0.7)

Notes: RA: Rectangular area (250 × 170 µm); D: Dark region; L: Light region; the numbers in parentheses are standard deviations.

**Figure 3:**
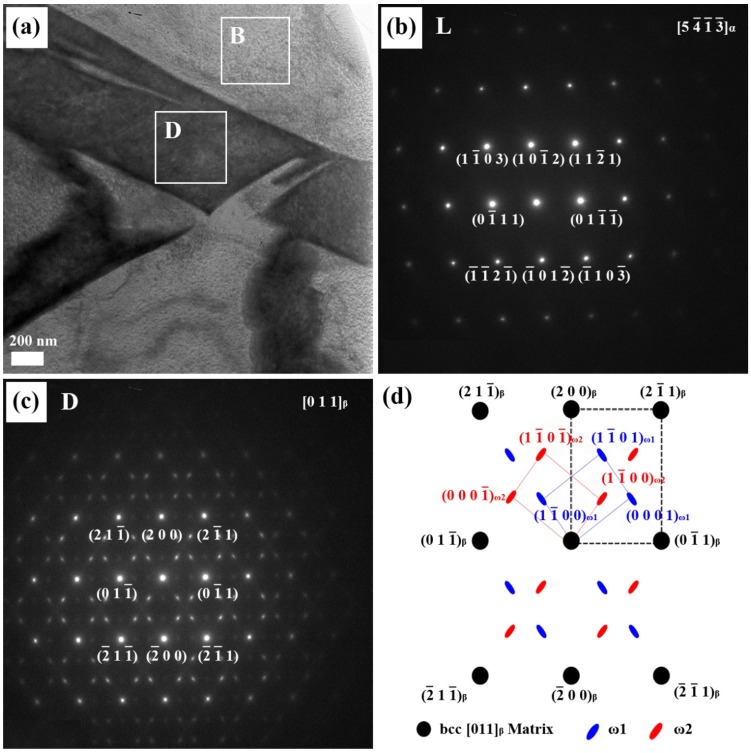
(**a**) The bright-field TEM image of the Ti-10Nb alloy; (**b**) the corresponding selected-area electron diffraction (SAED) patterns from the bright area (B); (**c**) the SAED patterns from the dark area (D); and (**d**) the key diagram of (**c**).

In order to understand the relationship between the microstructure and mechanical properties of Ti alloyed with Nb, microstructural observations were carried out using a high resolution transmission electron microscope (HR-TEM). As Nb content increased in the Ti-xNb alloys, the proportion of the α phase decreased gradually. The Ti-xNb alloys (x = 10, 15, and 20 wt %) were comprised of α, β, and ω phases, while the Ti-5Nb alloy was comprised of α and β phases. Although the ω phase was hardly detected by XRD, it was easily identified by TEM. The typical bright-field TEM image of the Ti-10Nb alloy, along with SAED patterns obtained from respective phases, is shown in Figure 3. Similar morphology to the Ti-10Nb alloy was also observed in the Ti-15Nb alloy. The bright regions in Figure 3a correspond to the α phase. The corresponding SAED pattern is shown in Figure 3b, which could be indexed in terms of $\left[5\overset{-}{4}\overset{-}{1}\overset{-}{3}\right]$_α_ zone axis of a hexagonal structure. The dark area represents the Nb-rich phase. The corresponding SAED pattern is shown in Figure 3c. A strong spot in the corresponding SAED pattern is obtained from the [011]_β_ zone axis of the matrix phase. Apart from the β phase spots, several weak diffuse diffraction spots were obtained from $\left[11\overset{-}{2}0\right]$]_ω_ zone axis; one set was indexed as ω_1_ and the other was assigned as ω_2_ [18]. The orientation relationship between the ω phase and the β phase was thus deduced as $\left[11\overset{-}{2}0\right]$_ω_//[011]_β_.

**Figure 4:**
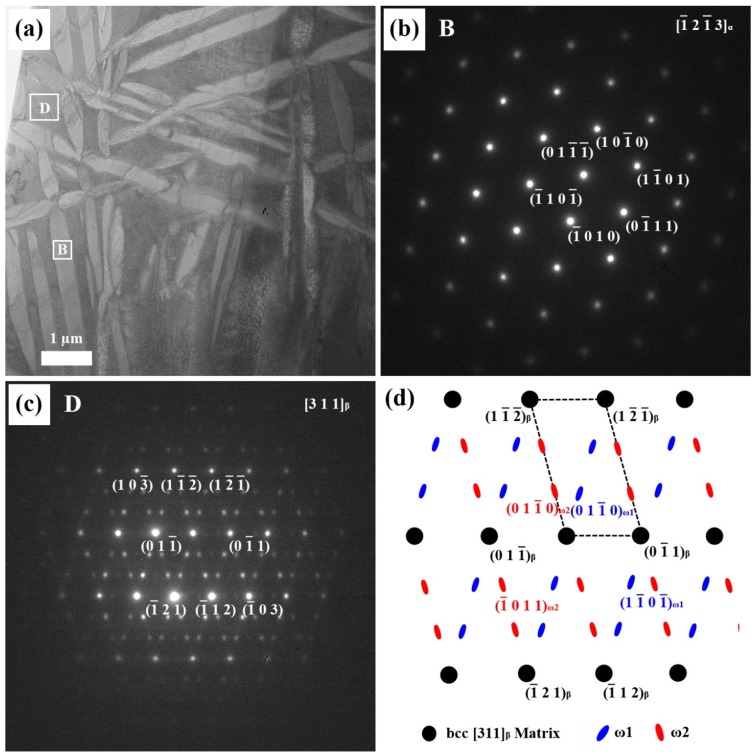
(**a**) The bright-field TEM image of the Ti-20Nb alloy; (**b**) corresponding selected-area electron diffraction (SAED) pattern from the bright area (B); (**c**) SAED pattern from the dark area (D); and (**d**) the key diagram of (**c**).

Similar behavior was observed in the Ti-20Nb alloy, as shown in Figure 4. In this case, the orientation of the α phase in Ti-20Nb was close to $\left[\overset{-}{1}2\overset{-}{1}3\right]$]_α_ as shown in Figure 4b. The dark area in Figure 4a represents the Nb-rich phase, and its corresponding SAED patterns were obtained from the [311]_β_ zone axis and $\left[2\overset{-}{1}\overset{-}{1}3\right]$_ω_ zone axis. The ω spots were fainter and more diffuse than the β spots. The observed orientation relationship was $\left[2\overset{-}{1}\overset{-}{1}3\right]$_ω_//[311]_β_, which is consistent with previous reports [19,20]. 

### 2.2. Effects of Nb Concentration on the Mechanical Properties

Phase transformation behaviors of the Ti-xNb alloys were measured by using differential scanning calorimetry (DSC). It was found that Nb addition was effective in decreasing the β-transus temperature. In the case of pure Ti, the phase transformation from α to β phase occurred at about 882 °C. The β-transus temperature of the Ti-15Nb alloy was 777 °C, whereas that of Ti-20Nb alloys was 734 °C. This result implies that Nb addition to the Ti alloy acted as a β stabilizer, thus lowering the β-transus temperature. 

We investigated the effects of alloying Nb on the mechanical properties and corrosion behavior of Ti. Table 2 shows Vickers hardness and elastic modulus values for each Ti-xNb alloy in comparison with those of cp-Ti. All of the Ti-xNb alloys had higher Vickers hardness values (*p* < 0.05) than that of cp-Ti (165 VHN). The Ti-5Nb alloy with α + β phases exhibited a hardness value of 358 VHN, higher than that of cp-Ti due to the solid solution strengthening by Nb addition. As Nb content increased from 5 to 10 wt %, the Ti-10Nb alloy showed decreased hardness, since the fraction of the β phase decreased in the α + β phases. The reduction of the β phase in Ti-10Nb alloy was an unexpected result. This point would need further study to examine the phase transition under various conditions of cooling after heat treatment. The Ti-15Nb alloy exhibited the highest hardness value of 413 VHN. This increase in hardness could be attributed to the increase in the β phase and the strengthening effect of the ω phase [16]. A similar result was observed in the study by Lee *et al.* [21]. When Nb content increased from 15 wt % to 20 wt %, the hardness of the Ti-20Nb alloy decreased to 332 VHN. In addition to high strength, a low modulus is essential for many load-bearing implant materials, because using implant materials with lower moduli (closer to that of human bone) can reduce the stress shielding effect [22]. Compared to the elastic modulus of the cp-Ti, that of the Ti-5Nb alloy was reduced due to the presence of the β phase. The increase in the elastic modulus of the Ti-10Nb alloy was related to the appearance of the ω phase in the microstructure. The values for Ti-xNb (x = 5, 10, 15, and 20) alloys were in the range of 114–149 GPa. Among the Ti-xNb alloys tested, Ti-10Nb and Ti-15Nb exhibited the highest and the lowest elastic modulus, respectively.

**Table 2:** Vickers hardness and elastic modulus values of Ti-xNb alloys and cp-Ti (*n* = 5).

Alloy Code	Vickers Hardness ± SD * (VHN)	Elastic Modulus ± SD (GPa)
cp-Ti	165 ± 4 ^a^*	132 ± 12 ^a^
Ti-5Nb	358 ± 27 ^b^	127 ± 7 ^c^
Ti-10Nb	338 ± 24 ^c^	149 ± 6 ^a^
Ti-15Nb	413 ± 21 ^a^	114 ± 5 ^b^
Ti-20Nb	332 ± 13 ^c^	127 ± 5 ^a^

Notes: * Within the same column, mean values with the same superscript alphabet are not statistically different (*p* < 0.05, Duncan post hoc grouping: a < b < c). SD means standard deviation.

For clinical use in dentistry, alloys must be able to bond to dental porcelains. It is known that Ti reacts strongly with gaseous elements such as oxygen at high temperature. The resultant Ti produces an excessively thick layer of TiO_2_, resulting in inadequate metal-ceramic bond strength [23]. The mismatch of the thermal expansion coefficient of titanium and ceramic may also affect the bond strength of the titanium ceramic system [23]. For proper bonding, the thickness of the metal oxide layer should be controlled and the thermal expansion coefficient of titanium should be compatible with that of the ceramic.

**Figure 5:**
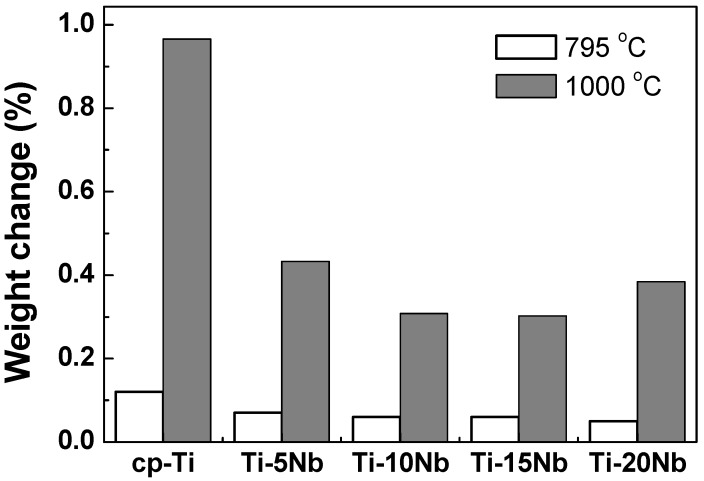
TGA analysis of cp-Ti and Ti-xNb alloys showing various degrees of weight gain (%) by heating in air.

The oxidation behavior of Ti-xNb alloys was assessed using thermogravimetric analysis (TGA). Figure 5 shows the result of the TGA experiment when the cp-Ti and Ti-xNb alloys were heated up to 795 °C and 1000 °C at a heating rate of 10 °C/min in air. Each of the samples was oxidized, and the weight gain was compared with that of the non-oxidized samples. All of the samples showed a single parabolic increase in mass during oxidation. All of the cp-Ti and Ti-xNb alloys had low mass changes (0.008–0.01 wt %) from room temperature to 580 °C. At a temperature higher than 580 °C, both cp-Ti and Ti-xNb alloys were rapidly oxidized, resulting in a significant increase in mass. All of the Nb containing samples showed a weight gain of about 0.1%–0.2% at a temperature of 795 °C, whereas at a temperature of 1000 °C, significant increases in mass of 0.4%–0.5% were observed. However, the final changes in the mass of Ti-xNb alloys were significantly less than that for the oxidation of cp-Ti, indicating that Nb addition to cp-Ti may restrict the oxidation rate of the alloy, and that Ti-xNb alloys show high oxidation protection ability. Weight gain decreased as Nb content increased. 

Thermal expansion coefficients (TEC) of Ti alloys with 5 and 20 wt % of Nb content were measured in the temperature range of 30–500 °C and compared with that of cp-Ti. The TEC of the ceramic used in this experiment was 8.6 × 10^−6^ °C^−1^. The TEC of cp-Ti (9.5 × 10^−6^ °C^−1^, 30–500 °C) was in agreement with the previously reported value (9.9 × 10^−6^ °C^−1^, 25–550 °C) [24]. The TEC of Ti-xNb was slightly lower than that of cp-Ti. The TEC values of Ti-5Nb and Ti-20Nb alloys in the temperature range of 30–500 °C were 9.35 × 10^−6^ °C^−1^ and 9.37 × 10^−6^ °C^−1^, respectively. The value of 0.5 × 10^−6^ °C^−1^ has been proposed as the acceptable difference in TEC between two materials [25]. Thus, alloying Ti with Nb was effective in reducing internal stress because these alloys had lower thermal expansion coefficients than cp-Ti. 

**Figure 6:**
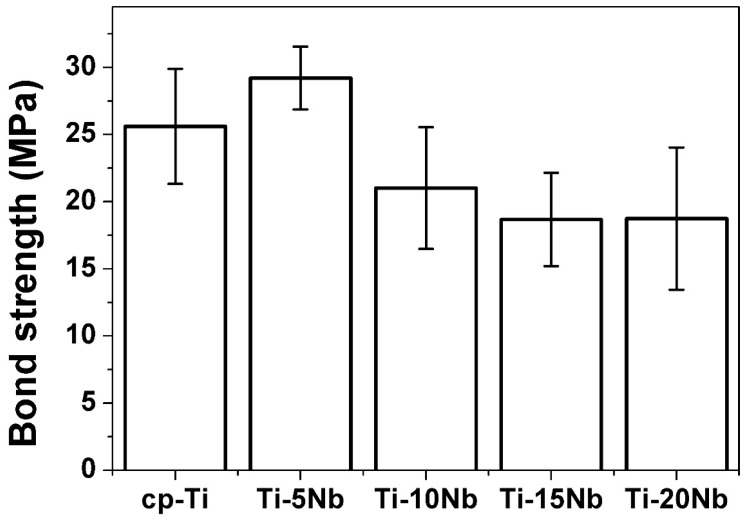
Bond strength of cp-Ti and Ti-xNb alloys to the ceramic (*n* = 5).

The bonding strengths of cp-Ti and Ti-xNb alloys to ceramic were investigated using a three-point bending test, and the results are shown in Figure 6. In the present study, the bond strength of cp-Ti was 25.6 MPa, which falls within the range of values reported by Pröbster *et al.* (21.4–34.0 MPa) [26]. Thus, the values determined in the present study can be considered to be reasonable. The bonding strength is affected by the characteristics of the surface oxide layer on cp-Ti and Ti-xNb alloys, and by the TEC value differences between substrate metal and ceramic coating material. The bond strength between Ti-5Nb and ceramic was 29.2 MPa, which was higher than the minimum value required according to the International Standard Organization (ISO) specification (25 MPa) [27]. The higher bond strength of Ti-5Nb could be attributed to the lower TEC difference between the Ti-5Nb alloy and the ceramic coating material. The bond strength of the Ti-xNb alloys tended to decrease as Nb content increased up to 15 wt % even though statistical significance was not found.

### 2.3. Effects of Nb Concentration on Corrosion Behavior

**Figure 7:**
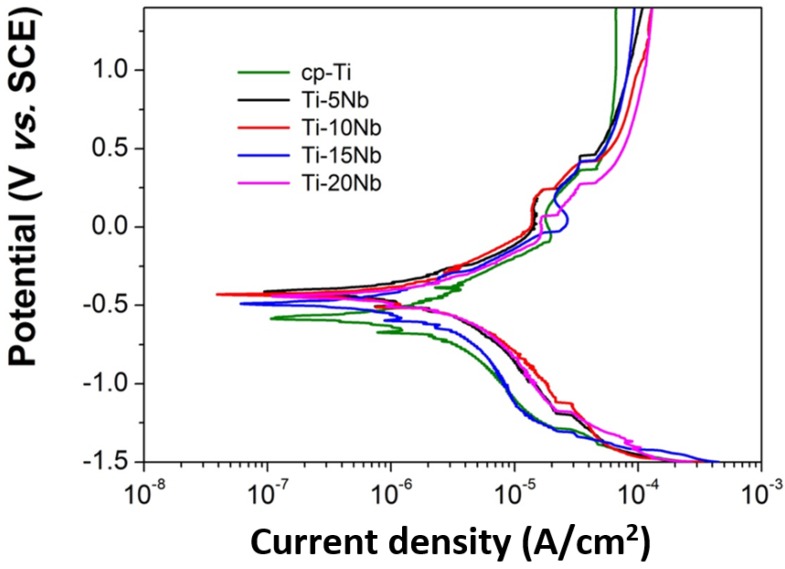
Representative potentiodynamic polarization curves of the cp-Ti and Ti-xNb alloys.

The corrosion behavior of the Ti-xNb alloys was evaluated using potentiodynamic polarization to investigate the effect of Nb content on the polarization curve in order to ascertain their suitability for dental implant applications. Potentiodynamic polarization curves of cp-Ti and Ti-xNb alloys were obtained in a potential range from –1.5 to +1.5 V in 0.9% NaCl solution and the results are shown in Figure 7. All curves exhibited similar general features. Cathodic branches exhibited a current density that decreased as the applied potential increased. This was attributed to hydrogen evolution and/or oxygen reduction on the electrode surface. Anodic branches exhibited an active-passive transition in all cases, with apparently small current density oscillation. These regions may be associated with the formation of one or more protective films. It is known that the addition of a small amount of Pt or Pd to Ti is very effective in improving the corrosion resistance of titanium due to promotion of the active-passive transition by the enhanced cathodic reaction [28]. The shift in the electrochemical potential of the Ti-xNb alloys could be explained in the same manner. Our experimental results showed a similar trend of a shift in electrochemical potential in the positive direction by addition of Nb to Ti, indicating a remarkable enhancement of corrosion resistance due to Nb addition [29].

Using the Tafel extrapolation method, corrosion parameters of cp-Ti and Ti-xNb alloys were calculated in both the anodic and cathodic branches of the potentiodynamic polarization curves (shown in Table 3). Table 3 shows the values of corrosion potential (*E_corr_*), corrosion current density (*I_corr_*), Tafel slopes (β*_a_* and β*_c_*), and corrosion rate (*C_R_*). The *E*_corr_ and *I_corr_* of cp-Ti were found to be approximately −0.575 V and 0.611 μA/cm^2^, respectively, which were in agreement with the previously reported values [30]. The corrosion rate of cp-Ti was 1.88 × 10^−2^ mm/year. Average corrosion potentials of the investigated Ti-xNb (5, 10, 15 wt % Nb) alloys were higher than that of cp-Ti (*p* < 0.05). Also, the addition of Nb to Ti resulted in the lowering of the passive current density (0.33–0.47 μA/cm^2^), which indicated the formation of a more protective passive film. The decreased *I_corr_* and increased *E_corr_* values in the positive direction for Ti-xNb alloys demonstrated a higher corrosion resistance than that of cp-Ti. Low corrosion rates were observed for all of the Ti-xNb alloys. Among the Ti-xNb alloys, Ti-10Nb displayed the greatest corrosion resistance with an *E_corr_* of −0.456 V, *I_corr_* of 0.33 μA/cm^2^, and *C_R_* of 0.964 × 10^−2^ mm/year.

**Table 3:** Corrosion potential (*E_corr_*) and corrosion current density (*I_corr_*) of cp-Ti and Ti-xNb alloys (*n* = 3).

Alloy Code	*E_corr_* (± SD) (V)	*I_corr_* (±SD) (μA/cm^2^)	β*_a_*	β*_c_*	*C_R_* (mm/year)
cp-Ti	−0.58 (0.04) ^a,^*	0.61(0.17) ^a^	0.27(0.09) ^a^	−0.19(0.02) ^b^	1.88(0.54) × 10^−2 b^
Ti-5Nb	−0.46 (0.07) ^a^	0.40 (0.06) ^a,b^	0.19(0.04) ^a^	−0.13(0.02) ^a^	1.20(0.19) × 10^−2 a^
Ti-10Nb	−0.46 (0.05) ^a^	0.33 (0.05) ^b^	0.18(0.02) ^a^	−0.14(0.04) ^a^	0.96(0.14) × 10^−2 a^
Ti-15Nb	−0.49 (0.09) ^a^	0.40 (0.03) ^a,b^	0.19(0.02) ^a^	−0.14(0.03) ^a,b^	1.13(0.09) × 10^−2 a^
Ti-20Nb	−0.52 (0.14) ^a^	0.47(0.16) ^a,b^	0.18(0.03) ^a^	−0.14(0.01) ^a^	1.31(0.46) × 10^−2 a,b^

Note: * Within the same column, mean values with the same superscript alphabet were not statically different at 5% (*p* < 0.05; a < b).

### 2.4. Cytotoxicity Based on the Agar Overlay Test and WST-1 Assay

**Figure 8:**
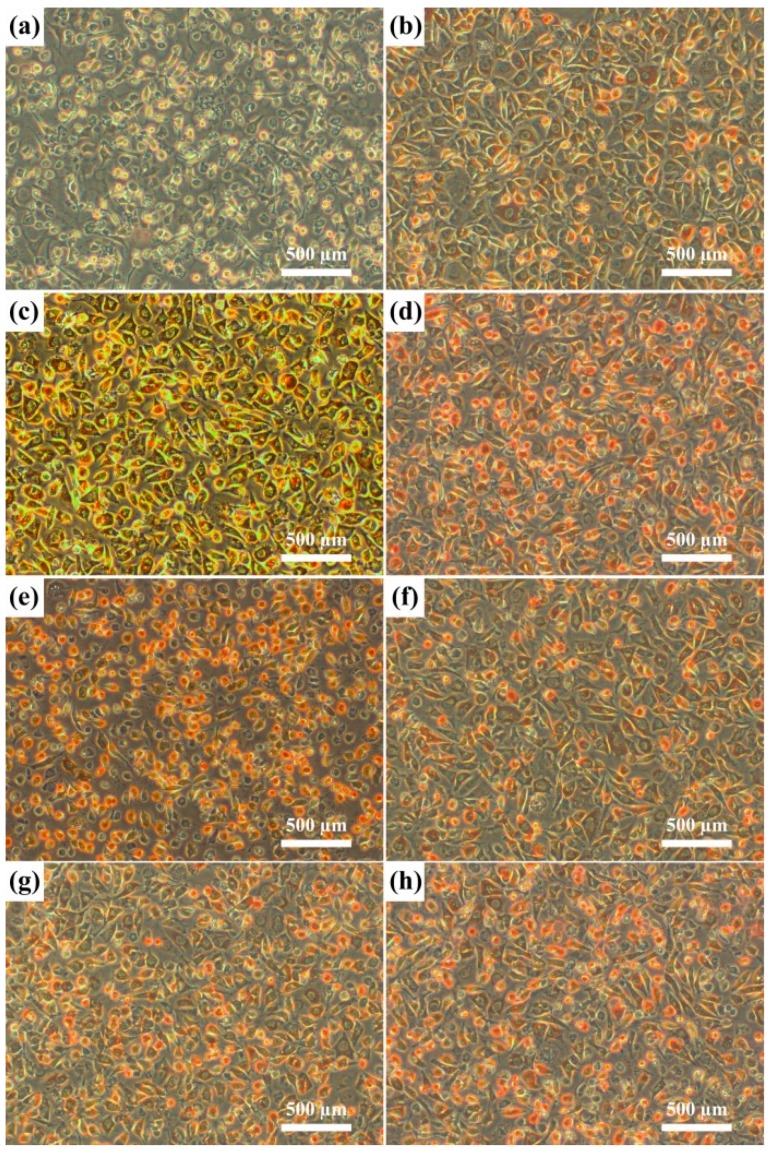
Photographs (×100) of the L929 cell line. (**a**) PU = polyurethane (positive control); (**b**) PE = polyethylene (negative control); (**c**) cp-Ti; (**d**) pure Nb; (**e**) Ti-5Nb; (**f**) Ti-10Nb; (**g**) Ti-15Nb; and (**h**) Ti-20Nb in the agar overlay test.

The results of the agar-overlay test are shown in Table 4. Five specimens were prepared for each material to test the cytotoxicity. Toxicity was evaluated by the size of the zone of decolorization (zone index) and the degree of cell lysis (lysis index). All of the Ti-xNb alloys and pure Nb specimens did not decolorize the neutral red stained cells in the agar overlay test (Figure 8). Therefore, based on the agar overlay test, cp-Ti and pure Nb were noncytotoxic with an average cell response of 0/0. The cytotoxicity of Ti-xNb alloys was also found to be unaltered by the Nb content in the alloy. Cell viability was determined by the water-soluble tetrazolium-1 (WST-1) assay. The samples are considered to be biocompatible if cell viability on the metal was equivalent to or greater than that of the control group. 

**Table 4:** Cytotoxicity of pure metal and Ti-xNb alloys based on the agar overlay test (*n* = 5).

Alloy Code	Decolorization Index (DI)	Lysis Index (LI)	Cell Response	Cytotoxicity
cp-Ti	0	0	0/0	none
Pure Nb	0	0	0/0	none
Positive control (polyurethane)	3	3	3/3	moderate
Negative control (polyethylene)	0	0	0/0	none
Ti-5Nb	0	0	0/0	none
Ti-10Nb	0	0	0/0	none
Ti-15Nb	0	0	0/0	none
Ti-20Nb	0	0	0/0	none

**Figure 9:**
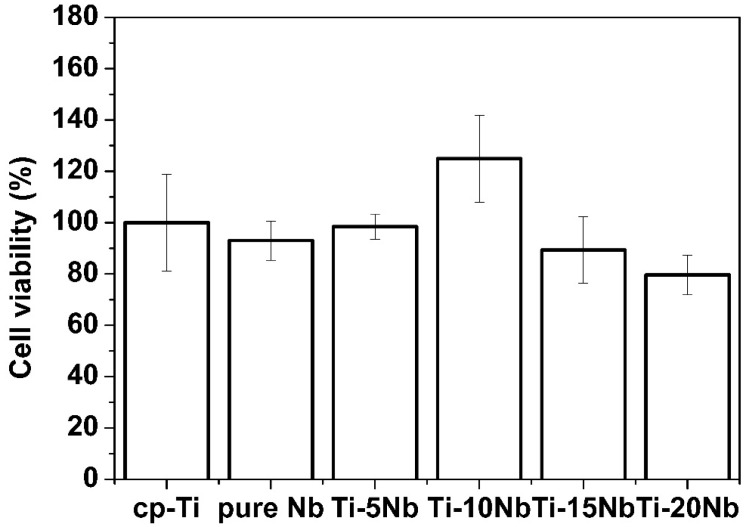
Percent cell viability of pure metals and their Ti based alloys *versus* cp-Ti after cell culture for 24 h (*n* = 7).

As shown in Figure 9, pure Nb demonstrated good biocompatibility as indicated by cell viability of 93.0% ± 7.6%, compared with that of the control group. This result is consistent with previous research [31]. Among the tested Ti-Nb alloys, the Ti-10Nb alloy showed the greatest cell viability at 124.9% ± 16.9%, which was greater than that of cp-Ti. The high cell viability of the Ti-10Nb alloy could be attributed to its excellent corrosion resistance. From electrochemical measurements, corrosion resistance increased by alloying Ti with Nb. Even though the Ti-10Nb had the best corrosion resistance among tested alloys, the differences between corrosion resistance values of Ti-xNb alloys were not significant (Table 3). With an Nb content of 5 wt %, cell viability was also comparable to that of cp-Ti (*p* > 0.05).

## 3. Experimental Section

### 3.1. Materials and Preparation

Ti-Nb alloys (5, 10, 15, and 20 wt % Nb) were prepared by arc-melting the constituent elements on a water-cooled copper hearth using a tungsten electrode under a high-purity argon atmosphere. The starting materials (Ti sponge, Alfa Aesar, Ward Hill, MA, USA, 99.95%; Nb ingot, LS-Nikko Co. Inc., Seoul, Korea, 99.95%) were used without further purification. During the arc-melting procedure, a titanium getter was heated prior to melting the reactant mixture to further purify the argon atmosphere. The samples were remelted several times to promote sample homogeneity. Subsequently, the samples were heat treated using a tube furnace under an argon atmosphere (99.9999%) for 4 h at a temperature 150 °C lower than their respective solidus temperatures. The samples were then cooled to 600 °C in a furnace at a rate of 10 °C/min and air-cooled to room temperature [32]. 

### 3.2. Phase Analysis and Microstructural Characterization

Phase analysis and structural characterization were performed by XRD. The XRD diffraction patterns were collected for the bulk sample using a X’Pert PRO Multi Purpose X-Ray Diffractometer (40 kV and 40 mA) with Cu K_α_ (λ = 1.54056 Å). The scanning speed was 2°/min and the scanning angle ranged from 20° to 80° 2θ. The cp-Ti sample was used as a control. The lattice parameters were obtained by least squares refinement of data in the 2θ range of 20°–80° with the aid of a Rietveld refinement program [33]. The phase transformation of Ti-xNb alloys was investigated by heating approximately 200 mg of the sample to 1000 °C at a rate of 20 °C/min using a differential scanning calorimeter (DSC, DSC 404 C, Netzsch, Selb, Germany). For metallographic examination, the sample surfaces were mechanically abraded using 320–4000 grit SiC papers, followed by abrading with Al_2_O_3_ particles of 5, 1, and 0.3 µm in size. The abraded samples were sequentially washed with acetone, ethanol, and distilled water. After etching with Keller’s solution (H_2_O: 65% HNO_3_: 32% HCl: 40% HF = 95: 2.5: 1.5: 0.5), the metallurgical structures were examined using SEM (Hitachi, S-3000N, Tokyo, Japan) and EDX (EMAX, Horiba, Kyoto, Japan). Phase identification by HR-TEM (Philips, Technai-F20, Amsterdam, The Netherlands) and SAED was performed on selected parts of the samples.

### 3.3. Measurement of Mechanical Properties

The surface of cp-Ti and Ti-xNb alloys were polished using 320–600 grit SiC papers. The microhardness of each sample was measured five times in the center of the sample using a Vickers microhardness tester (Zwick, Postfach4350, Ulm, Germany) with a 500-g load for 30 s. After the microhardness test, the samples were polished again up to #600 grit. Elastic modulus testing for each sample was performed five times in the center of the sample using a Nanoindenter XP (MTS Co., Dubuque, IA, USA) with a Berkovich diamond indenter and analyzed with the continuous stiffness measurement technique. The maximum indentation depth was 2 μm. The oxidation behavior of Ti-xNb alloys was tested by TGA (SDTA 851e, Mettler-Toledo, Columbus, OH, USA), which measures the change in mass caused by oxidation. Samples of 4.5 mm × 4.2 mm × 14.0 mm in size were heated to 795 °C or 1000 °C at a heating rate of 10 °C/min with a 50 mL/min air flow rate. Thermal expansion coefficients were obtained from the temperature-dependent change in the length of the material measured using a dilatometer (L75 PT1500, Linseis GmbH, Selb, Germany) from 30 °C to 1000 °C in an Ar atmosphere. Values were continuously recorded using a heating rate of 5 °C/min. Linear thermal expansion coefficients were determined in the temperature range from 30 °C to 500 °C. Bond strength testing was performed with a 3-point bending test on a universal testing machine (Instron 4302; Instron Co., Ltd., Norwood, MA, USA) according to ISO specifications [27]. For this investigation, low fusing porcelain (Triceram^®^, Dentaurum, Pforzheim, Germany) was used. The porcelain-veneered specimens were heated in a furnace (EsGAiA, J. Morita Co., Kyoto, Japan) according to the manufacturer's instructions. Bond strength (τ_b_) was calculated using the following equation:

(1) τ_b_ = k · F (N/mm^2^)

where, F is the maximum force applied in Newtons before debonding (failure load), and k is a constant determined from the thickness of the metal substrate and the elastic modulus of the metallic material [27].

### 3.4. Electrochemical Analysis

To observe corrosion behavior, potentiodynamic anodic polarization tests were conducted at a scan rate of 5 mV/s from −1.5 V to +1.5 V (SCE) using a potentiostat (WAT100, WonA Tech Co., Ltd., Seoul, Korea) in a 0.9% NaCl solution at 37 ± 1 °C. At least three samples were tested to confirm the experimental results. The surface of the sample with a 10 mm diameter was mechanically polished with SiC paper up to #2000 grit. Electrochemical measurements were recorded using the three electrode technique consisting of the working electrode (test samples), the counter electrode (high density carbon), and the reference electrode (saturated calomel electrode) [34]. Before immersing the test sample, the electrolyte was bubbled with Ar gas at 150 mL/min for more than 20 min to eliminate the residual oxygen. The used electrolyte was replaced with a fresh electrolyte before each measurement. The exposed surface area of samples in the electrolyte was 0.283 cm^2^. Potentiodynamic polarization curves were plotted using an automatic data acquisition system. Both corrosion potential and current density were estimated by Tafel plots using both anodic and cathodic branches.

The corrosion rate (*C_R_*; metal dissolution rate), in millimeters per year, was calculated using the following standard equation:

(2) *C_R_* = (*I_corr_* × *K* × *E_w_*)/(*A* × *d*)

where, *K*, *E_w_*, and *d* are the characteristic properties of each sample. *K* is the corrosion rate constant (3272 mm/year), *E_w_* is equivalent weight, *A* is the area, and *d* is the density [35].

### 3.5. Cytotoxicity Test

The biocompatibility of the studied Ti-xNb alloys and cp-Ti was determined by evaluating cytotoxicity based on the WST-1 assay and agar overlay test. Seven samples, each with a diameter of 10 mm, were prepared for each Ti-xNb alloy and cp-Ti. The sterilized sample slices were placed in 48-well plates. Cell viability was determined using a colorimetric assay based on the cleavage of tetrazolium salt WST-1 (Roche, Mannheim, Germany) by mitochondrial dehydrogenases. The resulting formazan dye that decomposed from tetrazolium salts was quantified by a spectrophotometer (Microplate reader, Bio-Rad, Hercules, CA, USA) and was directly correlated with the number of metabolically active cells in the culture to measure cell viability. 

For the WST-1 assay, L-929 mouse fibroblast cells were cultured in a 100 mm-diameter Petri dish using 10 mL RPMI 1640 culture medium supplemented with 10% FBS prior to the cytotoxicity assay. 100 μL of the cell suspension containing 5 × 10^4^ cells/mL was seeded on the test samples in 48-well plates and incubated at 37 °C under a 5% CO_2_ atmosphere for 24 h. The cells were detached from the test samples with Trypsin-EDTA solution, the sample discs were removed from the media, and 100 μL RPMI 1640 was added to each well. Then, 100 μL of the mixture was transferred to a 96-well culture plate, and the cells were incubated with 10 μL WST-1 solution at 37 °C and 5% CO_2_ for 4 h. Absorbance for each well was measured at 450 nm using a spectrophotometer. 

For the agar overlay test, 10 mL of L-929 cell suspensions containing 3 × 10^5^ cell/mL were seeded in cell culture dishes of 100 mm in diameter, and incubated to confluence for 24 h at 37 °C and 5% CO_2_. After the incubation process, the supernatant culture medium was replaced with 10 mL of freshly prepared agar medium containing RPMI 1640, 5% FBS, and 3% agarose mixture. After the agar layer was solidified, the agar medium was stained with 10 mL of neutral red solution (0.01% in phosphate-buffered saline, SIGMA, St. Louis, MO, USA) for 15 min at room temperature in the dark. Excess dye was then removed, and the test specimens were placed on the agar surface. The positive (0.25% zinc dibutyldithiocarbamate (ZDBC) polyurethane film) and negative (polyethylene sheet) controls were tested along with the test samples. All of the prepared dishes were subsequently incubated for 24 h at 37 °C under a 5% CO_2_ atmosphere. The cytotoxic effects were examined using a microscope by evaluating the decolorized zones and cell lysis around and/or under the specimens according to ISO7405 [36]. Each test was repeated five times. The decolorization zone index (ZI) was detected using the grade of the decolorizing cell zone and was scored as follows: (0) no decolorization detectable; (1) decolorization only under the specimen; (2) decolorization zone not greater than 5 mm from the specimen; (3) decolorization zone not greater than 10 mm from the specimen; (4) decolorization zone greater than 10 mm from the specimen; and (5) the total culture was decolorized. The cell lysis index (LI) was defined as a loss of cell membrane integrity and was scored as follows: (0) no cell lysis detectable; (1) less than 20% cell lysis; (2) 20%–40% cell lysis; (3) >40%–<60% cell lysis; (4) 60%–80% cell lysis; and (5) more than 80% cell lysis. Scoring for the cytotoxicity of the samples was based on the median scores of the decolorization zone index and the lysis index, and was represented by the cell response (ZI/LI). Cytotoxicity was classified as follows: noncytotoxic with a cell response of 0/0; mildly cytotoxic with a cell response of 1/1; moderately cytotoxic with cell responses of 2/3–3/3; and markedly cytotoxic with cell responses of 4/4–5/5.

## 4. Conclusions

Experimental results indicated that both the microstructure and mechanical properties of the cast Ti-xNb alloys were sensitive to Nb content. Based on the results of XRD and optical microscopy, the Ti-5Nb alloy consisted of α and β phases. The ω phase was observed in Ti-xNb alloys (x = 10~20 wt %) with α + β phases, and the ω phase increased as Nb content increased. The β and ω phases influenced increases in Vickers hardness. The β phase caused a decrease in the elastic modulus, while the ω phase increased the elastic modulus. The cast Ti-xNb alloy exhibited better oxidation protection ability than cp-Ti in high temperatures. High temperature oxidation behavior was influenced by the composition of the α + β phases. The differences in TEC values between the ceramic coating and the Ti-xNb alloys were smaller than that between the ceramic coating and the cp-Ti. Alloying Ti with Nb may be effective in reducing internal stress due to lower TEC values than that of cp-Ti. The Ti-5Nb alloy had bond strength that is sufficient for clinical use, and all Ti-xNb alloys showed better corrosion resistance than cp-Ti. Based on the agar overlay test, all of the Ti-xNb alloys were noncytotoxic, and the Ti-10Nb alloy demonstrated good biocompatibility as indicated by a cell viability of 124.9% ± 16.9%. 
